# A Brief Mobile Evaluative Conditioning App to Reduce Body Dissatisfaction? A Pilot Study in University Women

**DOI:** 10.3389/fpsyg.2019.02594

**Published:** 2019-11-21

**Authors:** Thierry Kosinski

**Affiliations:** Univ. Lille, EA 4072 – PSITEC – Psychologie : Interactions, Temps, Emotions, Cognition, Lille, France

**Keywords:** evaluative conditioning, app, exposure, body dissatisfaction, prevention

## Abstract

**Objective:**

Body dissatisfaction is a major risk factor underlying vulnerability to eating disorders. Experimental studies conducted in controlled environments suggest that body dissatisfaction could be improved by using evaluative conditioning (EC). The present study evaluates the feasibility of using an EC app in everyday life and the effects of its use on body dissatisfaction.

**Method:**

We designed a game-like app inspired by the Therapeutic EC app. 60 participants were randomly assigned to two conditions. Participants in the EC condition had to pair photographs of their own body with positive photographs, while participants in the neutral condition had to pair photographs of their own body with neutral photographs. We tested the effect of use of the app on body dissatisfaction, drive for thinness, self-esteem, depressive symptoms and eating behaviors.

**Results:**

Analysis revealed that participants in all conditions presented a significant decrease in body dissatisfaction, drive for thinness and an increase in self-esteem. However, contrary to our hypothesis, these effects were no greater in the EC condition than in the neutral condition.

**Conclusion:**

This is the first study to evaluate the effects of an app-based EC intervention targeting body image. Results appear to be promising and the app could easily be implemented in an ecological setting as it is a low effort, attractive and accessible intervention. However, the findings question the idea that EC was responsible for the observed effects which could be explained by the exposure effect. Results are discussed.

## Introduction

Body dissatisfaction consists of dysfunctional, negative beliefs and feelings about one’s weight and shape (Garner, 2002). Body dissatisfaction is a widespread and stable phenomenon across the population, particularly in women. Body dissatisfaction is studied because it is associated with numerous negative outcomes and is considered a major risk factor for various psychopathologies (Stice et al., 2011). As a consequence, eating disorder prevention programs address, among others, body dissatisfaction as part of their content (for a review see Littleton and Ollendick, 2003).

Many processes may lead to body dissatisfaction. For example, by conveying a thin ideal of beauty, the socio-cultural environment (family, peers, and the media) may contribute to the development and maintenance of body dissatisfaction (Thompson and Heinberg, 1999). Women who do not achieve ideal thinness would perceive this as a failure resulting in body dissatisfaction (Myers and Crowther, 2007). Body dissatisfaction could also result from social comparison, when the comparison is unfavorable (Tiggemann and McGill, 2004). Lastly, the discrepancy between the current body and an ideal body may lead to rumination about weight and shape, which in turn maintains body dissatisfaction (Etu and Gray, 2010; Maraldo et al., 2016). These different processes may lead to recurrent negative thinking about one own’s body.

Various studies have examined how body dissatisfaction could be improved. Among these studies, experimental research conducted in laboratory settings suggests that body dissatisfaction could be improved by using evaluative conditioning (EC).

Evaluative conditioning can be considered a form of pavlovian conditioning (PC). PC corresponds to a change in behavior that results from the pairing of an unconditioned stimulus (US, e.g., electric shock) with a conditioned stimulus (CS, e.g., light). This pairing results in a change in behavior in response to the CS (e.g., defensive preparatory response). The same methodology (CS-US pairing) is used to study EC. The only difference between these two forms of learning is that EC procedures are only interested in the changes in appreciation of the CS. The EC effect is defined as a change in the pleasantness of a conditioned stimulus (CS) resulting from its association with a valenced stimulus (unconditioned stimulus; US) (De Houwer, 2007).

Evaluative conditioning is now considered a meaningful way to create and change implicit and explicit evaluations. One important property of EC is its resistance to extinction. Whereas most conditioned responses are typically reduced by presentations of the CS without the US, several studies have shown that EC is unaffected by unreinforced CS presentations (e.g., Hermans et al., 2002). In other words, when a stimulus has become negatively perceived through association with a negative US, it will remain so, even if it is no longer paired with the US. While there are still some debates regarding the mechanisms underlying the EC effect, there is strong support for the idea that EC depends on non-automatic processes. Indeed, EC has been shown to depend on the memory of the CS-US pairing (contingency awareness), attention, goal, and cognitive resources (Gast et al., 2012). EC has been demonstrated with a wide range of stimuli (e.g., pictures, odors, and flavors) in many fields of psychology (e.g., consumer science, social psychology, and learning psychology) with a variety of procedures.

Recently, EC has been used to study clinical phenomena like body dissatisfaction, in order to change problematic acquired valences. Jansen et al. (2008) proposed that negative thinking related to the body can be considered an US and the body can be considered a CS. Thus, recurrent negative thinking about one own’s body, or negative feedback about body shape could result in multiple associations between one’s body and negative information resulting in an EC effect. Thus, theoretically, one could change a negative evaluation of one’s body through new associations with positive stimuli (counter-conditioning).

Martijn et al. (2010) used a computerized game in which the participant’s task was to click on a photograph of a body presented at one of the four corners of the screen. Some photographs were of unknown people, others corresponded to the participants’ own body (CS). In the experimental condition, when participants clicked on a photograph of their own body, a smiling face (US) appeared on the computer screen. They observed that women with high body concern demonstrated a decrease in body dissatisfaction and an increase in self-esteem. More recently, in a replication study, Glashouwer et al. (2019) found similar results. After the training in the experimental condition, participants rated pictures of their own body as more positive than participants in the control condition. However, the EC effect was only present for these pictures and did not transfer to self-report on body satisfaction. Moreover, women with high and low body concern did not benefit differentially from the EC. Finally, Aspen et al. (2015) observed that such a procedure also allowed a decrease in weight and shape concern and a reduction in self-reported restrictive eating in women with high body dissatisfaction. These changes were maintained at 12-week follow-up.

These results are particularly encouraging as EC procedures seem to be effective in countering body dissatisfaction, at least in non-clinical samples. However, some limitations still need to be overcome. Indeed, apart from a study by Glashouwer et al. (2018) which used an online EC procedure on a clinical sample (and did not find an effect of the intervention), experimental interventions based on EC targeting body dissatisfaction are currently restricted to controlled laboratory situations (participants are trained in laboratories, they wear standardized clothes). Technologies allowing laboratory methodologies to be easily delivered in the everyday world are needed.

Recently, Franklin et al. (2016) tested a brief game-like mobile app, designed to decrease self-injurious behavior and increase self-esteem. Participants were trained to associate self-related words to positive pictures and self-injury related stimuli to negative pictures using their smartphones. Results showed that the EC procedure led to a reduction in self-injurious thoughts and behaviors. These findings suggest that EC can be effective in changing the evaluation of stimuli directly in the ecological environment of participants. Kollei et al. (2017) recently found evidence that an app-based intervention (using approach-avoidance training) may significantly reduce body dissatisfaction. The present experiment constituted a pilot study to test the possibility of using an app-based EC procedure to induce change in body dissatisfaction in a non-clinical sample. This is the first time that an app using EC is tested to counter body dissatisfaction.

## Materials and Methods

### Participants and Design

Participants were 60 French-speaking undergraduate women Mean Age = 19.55(1.36); Mean BMI = 22.09(4.39) from a French University. The design of the study included assessment time (pre-test vs. post-test) as a two-level within-subject factor and condition (EC vs. neutral) as a two-level between-subject factor. Participants were randomly assigned to either the neutral condition (*n* = 30) or the EC condition. Group assignments were predetermined by subject number, odd numbered participants were assigned to the EC condition, even numbered participants were assigned to the neutral condition. The procedure was in accordance with the World Medical Association’s Declaration of Helsinki and was approved by the local ethics committee (2017-01-57).

### Material

#### Unconditioned Stimuli (USs)

**FIGURE 1:**
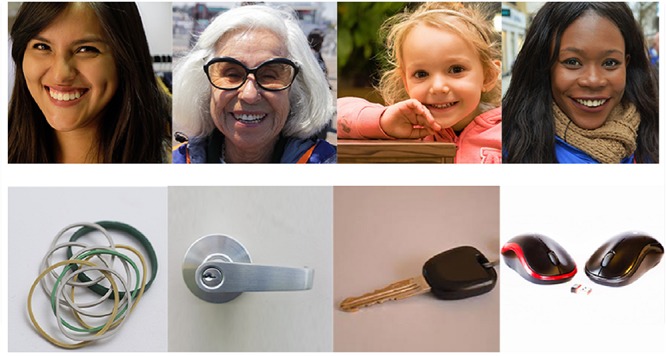
USs+examples on the top line, USsNexamples on the bottom line.

Body dissatisfaction is supposed to rely on one’s own evaluations, but also depends on the perceived approval of other people. Thus, previous experiments paired participants’ own bodies with smiling faces. We decided to use a methodology similar to those used in previous experiments. Our 21 USs consisted of positive photographs. Each US+ corresponded to smiling women’s faces of different ages and skin color. Each photograph had been pretested to (a) elicit a positive affective response and to (b) correspond to “ordinary people” and not to a feminine ideal (e.g., photoshopped models seen in advertising; see example Figure 1). This last choice was made to avoid the social comparison effect which could increase body dissatisfaction (Tiggemann and McGill, 2004).

#### Neutral Stimuli (NSs)

Our 21 NSs consisted of neutral photographs. These NSs corresponded to photographs of neutral objects from everyday life (e.g., keys, chair, door handles, and rubber bands). Each photograph had been selected from a pretest to (a) elicit a neutral affective response and to (b) not be related to eating or beauty (see example Figure 1).

All photographs were Creative Commons Zero images found on the Internet.

#### Conditioned Stimuli (CSs)

Three photographs of each participant were taken by experimenters and were used as CSs. One photograph was of the participant’s face, two photographs were of the participant’s full body (front and profile) wearing their own clothes.

#### Intervention

**FIGURE 2:**
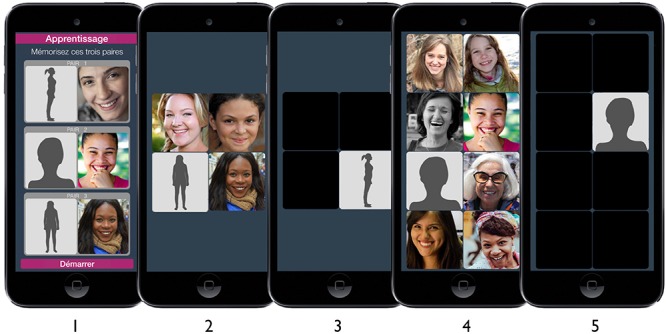
Screenshots of app for the EC condition. Shapes were replaced by participants pictures in the real app. Step 1: The sessions begin with a screen displaying pairs to learn. Step 2: Game session begins with 2 × 2 grid for the first 15 trials. Step 3: Alternative options are masked after the first pair member is selected for the second 15 trials. Step 4: Grid moves to a 2 × 4 grid for the third 15 trials. Step 5: Alternative options are masked after the first pair member is selected for the last 15 trials.

*For the EC condition*, we designed a brief game-like app, inspired by the app designed by Franklin et al. (2016). In our study, each game session began with the presentation of three different pairs of photographs that the participant was instructed to remember. Pairs were composed of one CS and one US+. CS-US+ associations were randomly determined by the app at the beginning of each session. During the game, at each trial, one of these three pairs appeared among distractor photographs. The participant’s task was to select these pairs as quickly as possible. Distractor photographs corresponded to other USs+. Each game session contained 60 trials. At the end of each trial, the participant received feedback about the accuracy of the response (a red or green screen for 100 ms). Every 15 trials, the game became harder as more distractors were added and other response options were blacked out when the first option was selected (see Figure 2). Points were awarded for fast and accurate responses; this score was displayed at the end of each session. Thus, at each trial, participants associated an image of their body and a specific US+ by successively selecting a photograph of their own body and the target US+ (sequential pairing). Moreover, each time a participant saw a photograph of their own body, this was accompanied by other USs+ (distractors) on the screen (simultaneous pairing). This constituted an EC procedure in which the participant’s body was paired with pleasant stimuli in several ways.

*For the neutral condition*, the same methodology was applied, however, NSs were used instead of positive stimuli (USs+).

#### Eating Disorder Inventory-2

The EDI-2 (Garner, 1991; French version: Criquillon-Doublet et al., 1995) measures eating disorder symptoms and associated psychological traits. In this study we focused on attitudes and behaviors concerning eating, weight, and shape. Thus, we only used the 23 items in the subscales measuring drive for thinness, bulimia and body dissatisfaction. These items were adapted to only take the last 7 days into account. In our sample, Cronbach’s Alpha (internal consistency) of the EDI-2 subscales drive for thinness and body dissatisfaction were, respectively 0.75 and 0.90 at pre-test and 0.81 and 0.92 at post-test. Cronbach’s Alpha for the bulimia subscale at post-test was 0.80.

#### Contour Drawing Rating Scale

The CDRS (Thompson and Gray, 1995) consists of nine drawings of a female figure (for female participants). Each drawing increases in size from extremely thin to very obese. Participants are asked to rate their ideal figure and their current size (perceived figure). The discrepancy between ideal and current size scores constitutes an index of body size dissatisfaction.

#### Eating Disorder Examination Questionnaire

The *EDE-Q* (Fairburn and Beglin, 1994; French version: Mobbs and Van der Linden, unpublished) measures eating disorder psychopathology over the last 28 days. In this study we only used the five items of the Restraint subscale. These items were adapted to only take the last 7 days into account. In our sample, Cronbach’s Alpha of EDE-Q was 0.85.

#### Center for Epidemiologic Studies Depression

The CES-D (Radloff, 1977; French version: Bouvard et al., 2013) consists of 20 items that measure depressive symptomatology in the general population. In our sample, Cronbach’s Alpha of CES-D at pre-test and post-test were, respectively 0.92 and 0.94.

#### Rosenberg Self-Esteem Scale

The RSES (Rosenberg, 1965; French version: Vallieres and Vallerand, 1990) consists of ten items that measure both positive and negative feelings about the self. In our sample, Cronbach’s Alpha of RSES at pre-test and post-test were, respectively 0.91 and 0.92.

### Procedure

Each participant filled in a consent form before the experiment. All participants were tested individually. The experiment began with pre-test assessments. Participants completed the Drive for Thinness and Body Dissatisfaction subscales of the EDI-2, the CDRS, the CESD and the RSES. Participants were then randomly assigned to one of the experimental conditions. The experimenter took the three photographs used as CSs directly within the EC app installed on an iPod Touch that was lent to the participant. The experiment was presented as dealing with the relationship between emotional state and performance in a self-related visual memory game (no reference to body dissatisfaction or eating behavior was included in the presentation of the study). Participants were asked to fulfill at least one game session a day for the next 7 days and the date for the post-test was scheduled about a week later. At post-test, participants completed the same evaluations as at pre-test in addition to the Bulimia subscale of the EDI-2 and the Restraint subscale of the EDE-Q.

## Results

### Analytic Plan

In order to ascertain the absence of differences between the two groups before the intervention, pre-test ratings of all measurements were subjected to independent-sample comparison. Then, for each participant, we computed five *evolution scores* corresponding to the difference between post-test and pre-test scores for the Drive for Thinness and Body Dissatisfaction subscales of the EDI-2, the CDRS, the CESD and the RSES. Negative differences corresponded to a reduction of the score from pre-test to post-test (see Table 1). These evolution scores, as well as the scores for the Bulimia subscale of the EDI-2 and the Restraint subscale of the EDE-Q (only assessed at post-test) and data from the app (e.g., number of sessions, number of correct responses) were analyzed using independent-sample comparison. Finally, evolution scores were analyzed to determine if they were significantly different from absence of evolution. In other words, we determined whether or not the differences between scores at pre-test and at post-test were statistically significant.

**TABLE 1:** Mean scores as function of condition and assessment time.

	**Pre-test assessment**		**Post-test assessment**		**Evolution score pre/post-test**	
	**EC condition M (SD)**	**Neutral condition M (SD)**	**EC condition M (SD)**	**Neutral condition M (SD)**	**EC condition M (SD)**	**Neutral condition M (SD)**
DT	22.80 (5.31)	22.43 (7.45)	19.23 (5.90)	19.00 (6.79)	−3.57(4.75)	−3.43(4.14)
BD	33.67 (8.87)	33.43 (10.98)	32.70 (9.32)	33.43 (10.98)	−0.97(4.26)	−1.87(6.56)
CDRS	−1.77(1.36)	−1.43(1.65)	−1.57(1.28)	−1.30(1.62)	−0.30(1.12)	−0.13(0.57)
CES-D	17.23 (9.48)	19.70 (11.54)	16.63 (11.94)	18.33 (11.17)	−0.60(9.02)	−1.37(7.97)
RSES	29.13 (5.42)	27.70 (6.51)	29.73 (5.49)	28.23 (6.66)	0.60 (4.19)	0.53 (2.06)
Bulimia	–	–	10.60 (4.77)	10.43 (3.27)	–	–
Restraint	–	–	7.47 (6.43)	7.70 (7.64)	–	–

*DT (drive for thinness), BD (body dissatisfaction), and Bulimia are three subscales of the Eating Disorder Inventory. CDRS = contour drawing rating scale. CES-D = center for epidemiologic studies depression. RSES = rosenberg self-esteem scale (RSES). Restraint is a subscale of the Eating Disorder Examination Questionnaire.*

Because normality of distribution was not respected, we used a non-parametric Mann-Whitney U test and a One-Sample Wilcoxon Median test as independent-sample comparison and one-sample comparison tests (comparison value = 0). No participants were excluded or dropped out, and there were no missing data.

### Pre-test Analysis and Application Check

Analysis of pre-test evaluations revealed no significant difference between the two conditions. In other words, Age (*U* = 422.5; *p* > 0.05), BMI (*U* = 460.00; *p* > 0.05), Drive for Thinness (*U* = 435.5; *p* > 0.05), and Body Dissatisfaction (*U* = 450.5; *p* > 0.05) subscales of the EDI-2, the CDRS (*U* = 493.5; *p* > 0.05), the CESD(*U* = 498.5; *p* > 0.05) and the RSES [*t*(58) = 0.927; *p*. > 0.05] were not different in the two conditions before the experimental manipulation.

On average, participants kept the app 10.00(5.38) days and completed 10.20(4.74) sessions during this period with an average of 55.81(3.07) correct responses (over 60 trials) per session. No difference between the two groups was observed on these variables (respectively, *U* = 371.5; *p* > 0.05; *U* = 520; *p* > 0.05; *p* > 0.05; *U* = 561.5; *p* > 0.05).

### Effect of Intervention

Independent comparison of evolution scores for body dissatisfaction revealed that participants in the EC condition did not elicit a stronger reduction in body dissatisfaction (*M* = −0.97) than participants in the neutral condition (*M* = −1.87; *U* = 427.5; *p* > 0.05) (Table 1). However, evolution of scores from pre to post-test, collapsed across conditions, revealed a reduction in body dissatisfaction. Indeed, participants elicited an evolution score significantly different from 0 (*W* = 419; *p* < 0.05) with a small effect size (*r* = 0.27) corresponding to a significant decrease in the body dissatisfaction score from pre-test to post-test independently of the condition.

Analysis of drive for thinness revealed the same pattern of results. Participants in the EC condition did not elicit a stronger reduction in drive for thinness (*M* = −3.57) than participants in the neutral condition (*M* = −3.43; *U* = 469.5; *p* > 0.05). However, overall, participants elicited an evolution score significantly different from 0 (*W* = 132.5; *p* < 0.001) with a large effect size (*r* = 0.67). In other words, all participants manifested a significant decrease in the drive for thinness score from pre-test to post-test, but this difference was not larger in the EC condition.

Analysis of self-esteem revealed the same pattern of results. Participants in the EC condition did not elicit a stronger augmentation in self-esteem (*M* = 0.60) than participants in the neutral condition (*M* = −0.53; *U* = 401; *p* > 0.05). However, overall, participants elicited an evolution score significantly different from 0 (*W* = 968; *p* < 0.05) with a small effect size (*r* = 0.29). In other words, all participants manifested a significant increase in self-esteem score from pre-test to post-test, but this difference was not larger in the EC condition.

Analysis of other scores revealed no effect regarding any other variables. Evolution scores for depressive symptoms (CES-D) and body size dissatisfaction (CDRS) where not different for the two conditions (respectively, *U* = 470.5; *p* > 0.05 and *U* = 429; *p* > 0.05). Moreover, these evolution scores were not significantly different from 0 (respectively, *W* = 574; *p* > 0.05 and *W* = 192; *p* > 0.05). Finally, bulimia and restraint scores were not different in the two conditions at the end of the experiment (respectively, *U* = 463; *p* > 0.05 and *U* = 477.5; *p* > 0.05). All data for this article can be found online (see Supplementary Table S1).

## Discussion

This study was designed to test the possibility of decreasing body dissatisfaction using EC within an app-based intervention. Participants used a brief EC game-like app during a week. Like Martijn et al. (2010) and Aspen et al. (2015) we observed a significant decrease in body dissatisfaction and an increase in self-esteem after the intervention. We also observed a decrease in drive for thinness. However, contrary to our hypothesis, these effects were not larger for the EC condition than for the neutral condition. This will be discussed. To the best of our knowledge, this is the first study to evaluate the effects of an app-based EC intervention targeting body image.

The current methodology was specifically developed to maximize the chances of obtaining an EC effect. The rules of each EC session requiring the participant to memorize pairs aimed to facilitate contingency awareness which has been found to be the most potent moderator of the EC effect (Hofmann et al., 2010).

In previous research observing an effect of an EC procedure, pictures of the participant’s body were followed by smiling faces (100%), while pictures of control bodies were followed by neutral (50%) or frowning faces (50%). These studies did not determine whether learning the *participant-positive* association, the *other-negative* association, or both together drove the effects. The *other-negative* association could in itself explain the effects observed. Indeed, Martijn et al. (2013) observed that when participants learned to associate pictures of others bodies (thin-ideal models) with negative stimuli, participants elicited an increase in body satisfaction scores. In the present research, no pictures of other people’s bodies were presented. Participants only learned to associate images of their own body with USs.

Given these precautions, our hypothesis was that any changes in body image related outcomes observed in participants in the EC condition would be driven by an EC effect relying on an association of images of the participants’ own bodies and USs. Participants would have learned to associate their own body with positive USs and elicited a reduction in body dissatisfaction. However, because the effects observed in the EC condition were not significantly different to those observed in the neutral condition, we cannot affirm that the EC effect was responsible for these outcomes.

The efficacy of the neutral condition could be explained by the exposure effect. In cognitive behavior therapy, exposure exercises usually require exposing participants to their own body (e.g., mirror exposure), in order to gradually extinguish negative responses to these stimuli. Body exposure has been shown to reduce body dissatisfaction as well as weight and shape concerns in women (Delinsky and Wilson, 2006). In the neutral condition, during each session, participants were exposed to images of their own body 60 times a day. The absence of superiority of the EC condition in the present study does not support the use of EC in its present form as a better intervention than mirror (or app-based) exposure in university women. Note that in Martijn et al. (2010), Aspen et al. (2015), and Glashouwer et al. (2019), the control condition consisted of randomly associating the participant’s body and other bodies with positive, neutral and negative stimuli which does not support the exposure explanation.

The present research does provide some contributions. The present study found that EC training could be implemented outside of the laboratory. Together with Kollei et al. (2017), our findings suggest that an app-based intervention may significantly reduce body dissatisfaction even if the present study did not support the idea that the EC effect was responsible for this change. Furthermore, previous research finding an influence of an EC procedure on body satisfaction only used standardized material (standard fitted black clothes). Here, we observed a decrease in body dissatisfaction with unstandardized photographs of participants wearing their own clothes. Given these points and that participants were not selected based on body concern, it is easy to imagine the implementation of this intervention as a real-life prevention program in a non-clinical population. Moreover, game-like methodologies (or gamification) facilitate the enjoyment and engagement of participants (Looyestyn et al., 2017), and should be encouraged in the development of mental health interventions.

While changes in body dissatisfaction, drive for thinness and self-esteem were observed, we found no evidence for a change of other measured outcomes (eating behaviors and depressive symptoms). These null-results could be explained by the nature of our population. Indeed, the only proof of the influence of an EC procedure on eating behavior was observed by Aspen et al. (2015) who only recruited participants considered at high risk for developing an eating disorder. Here, we did not recruit an at risk population, but typical university women. This could have resulted in a floor effect in relation to problematic eating behaviors. Finally, while depressive symptoms were not measured in previous studies, the same rationale could apply. Indeed, with the CES-D, the optimal cutoff for women was determined to be 23 (Henry et al., 2018), while in our sample, the mean score at pre-test assessment was 18,46. Thus, depressive symptoms could have been too low to be influenced by our method.

A number of limitations need to be considered when interpreting the present findings. As most previous studies, the present study was based on a sample composed of undergraduate women. Hence, the generalizability of the findings is limited. The absence of a control condition, without any task, or without exposure to photographs of the participant’s own body does not allow us to conclude with certainty regarding the reasons for the efficacy of the neutral condition. The present results are only based on self-report assessments which does not protect from a demand effect. Finally, despite our efforts in selecting USs, we can’t exclude the possibility that a social comparison effect occurred.

App based designs appear to be promising and could easily be implemented in the everyday world as they constitute low effort, attractive and accessible interventions. However, the present findings do not support the idea that EC is responsible for the observed effects which could be explained by the exposure effect. Future research should explore the effects of interventions on automatic evaluations and de-ambiguate the mechanisms involved.

## Data Availability Statement

The raw data supporting the conclusions of this manuscript are available through 10.6084/m9.figshare.8943302.v1.

## Ethics Statement

The studies involving human participants were reviewed and approved by the Comité d’Éthique pour la Recherche, Université de Lille (2017-01-57) https://www.univ-lille.fr/recherche/la-recherche-au-service-de-la-societe/ethiques/. The patients/participants provided their written informed consent to participate in this study.

## Author Contributions

TK designed the study, performed the experiments, analyzed the data, and wrote the manuscript.

## Conflict of Interest

The author declares that the research was conducted in the absence of any commercial or financial relationships that could be construed as a potential conflict of interest.

## References

[B1] Aspen V. Martijn C. Alleva J. M. Nagel J. Perret C. Purvis C. (2015). Decreasing body dissatisfaction using a brief conditioning intervention. *Behav. Res. Ther.* 69 93–99. 10.1016/j.brat.2015.04.003 25912670

[B2] Bouvard M. Denis A. Roulin J. L. (2013). Confirmation of the dimensions of the french version of the self-assessment scale of the center for epidemiological studies-depression (CES-D). *L’Encephale* 39 452–453. 10.1016/j.encep.2012.01.006 23347666

[B3] Criquillon-Doublet S. Divac S. Dardenne R. Guelfi J. D. (1995). “Le Eating Disorder Inventory,” in *Psychopathologie Quantitative*, eds Guelfi J. D. Gallac V. Dardenne R., (Paris: Masson), 249–260.

[B4] De Houwer J. (2007). A conceptual and theoretical analysis of evaluative conditioning. *Span. J. Psychol.* 10 230–241. 10.1017/S1138741600006491 17992949

[B5] Delinsky S. S. Wilson G. T. (2006). Mirror exposure for the treatment of body image disturbance. *Int. J. Eat. Disord.* 39 108–116. 10.1002/eat.20207 16231342

[B6] Etu S. F. Gray J. J. (2010). A preliminary investigation of the relationship between induced rumination and state body image dissatisfaction and anxiety. *Body Image* 7 82–85. 10.1016/j.bodyim.2009.09.004 19837639

[B7] Fairburn C. G. Beglin S. J. (1994). Assessment of eating disorders: interview or self-report questionnaire? *Int. J. Eat. Disord.* 16 363–370. 7866415

[B8] Franklin J. C. Fox K. R. Franklin C. R. Kleiman E. M. Ribeiro J. D. Jaroszewski A. C. (2016). A brief mobile app reduces nonsuicidal and suicidal self-injury: evidence from three randomized controlled trials. *J. Consult. Clin. Psychol.* 84:544. 10.1037/ccp0000093 27018530

[B9] Garner D. M. (1991). *Eating Disorder Inventory 2 Professional Manual.* Odessa, FL: Psychological Assessment Resources, Inc.

[B10] Garner D. M. (2002). “Body image and anorexia nervosa,” in *Body Image: A Handbook of Theory, Research, and Clinical Practice*, eds Cash T. F. Pruzinsky T., (New-York, NY: Guilford).

[B11] Gast A. Gawronski B. De Houwer J. (2012). Evaluative conditioning: recent developments and future directions. *Learn. Motiv.* 43 79–88. 10.1016/j.lmot.2012.06.004

[B12] Glashouwer K. A. Masselman I. de Jong P. J. (2019). Reducing body dissatisfaction by means of an evaluative conditioning procedure in undergraduate women: a replication study. *Behav. Res. Ther.* 121:103435. 10.1016/j.brat.2019.103435 31488242

[B13] Glashouwer K. A. Neimeijer R. A. de Koning M. L. Vestjens M. Martijn C. (2018). Evaluative conditioning as a body image intervention for adolescents with eating disorders. *J. Consult. Clin. Psychol.* 86:1046. 10.1037/ccp0000311 30507229

[B14] Henry S. K. Grant M. M. Cropsey K. L. (2018). Determining the optimal clinical cutoff on the CES-D for depression in a community corrections sample. *J. Affect. Disord.* 234 270–275. 10.1016/j.jad.2018.02.071 29554615

[B15] Hermans D. Vansteenwegen D. Crombez G. Baeyens F. Eelen P. (2002). Expectancy-learning and evaluative learning in human classical conditioning: affective priming as an indirect and unobtrusive measure of conditioned stimulus valence. *Behav. Res. Ther.* 40 217–234. 10.1016/S0005-7967(01)00006-7 11863234

[B16] Hofmann W. De Houwer J. Perugini M. Baeyens F. Crombez G. (2010). Evaluative conditioning in humans: a meta-analysis. *Psychol. Bull.* 136:390. 10.1037/a0018916 20438144

[B17] Jansen A. Bollen D. Tuschen-Caffier B. Roefs A. Tanghe A. Braet C. (2008). Mirror exposure reduces body dissatisfaction and anxiety in obese adolescents: a pilot study. *Appetite* 51 214–217. 10.1016/j.appet.2008.01.011 18342397

[B18] Kollei I. Lukas C. A. Loeber S. Berking M. (2017). An app-based blended intervention to reduce body dissatisfaction: a randomized controlled pilot study. *J. Consult. Clin. Psychol.* 85:1104. 10.1037/ccp0000246 28857576

[B19] Littleton H. L. Ollendick T. (2003). Negative body image and disordered eating behavior in children and adolescents: what places youth at risk and how can these problems be prevented? *Clin. Child Fam. Psychol. Rev.* 6 51–66. 10.1023/A:1022266017046 12659451

[B20] Looyestyn J. Kernot J. Boshoff K. Ryan J. Edney S. Maher C. (2017). Does gamification increase engagement with online programs? A systematic review. *PLoS One* 12:e0173403. 10.1371/journal.pone.0173403 28362821 PMC5376078

[B21] Maraldo T. M. Zhou W. Dowling J. Vander Wal J. S. (2016). Replication and extension of the dual pathway model of disordered eating: the role of fear of negative evaluation, suggestibility, rumination, and self-compassion. *Eat. Behav.* 23 187–194. 10.1016/j.eatbeh.2016.10.008 27816857

[B22] Martijn C. Sheeran P. Wesseldijk L. W. Merrick H. Webb T. L. Roefs A. (2013). Evaluative conditioning makes slim models less desirable as standards for comparison and increases body satisfaction. *Health Psychol.* 32:433. 10.1037/a0028592 22612560

[B23] Martijn C. Vanderlinden M. Roefs A. Huijding J. Jansen A. (2010). Increasing body satisfaction of body concerned women through evaluative conditioning using social stimuli. *Health Psychol.* 29 514–520. 10.1037/a0020770 20836606

[B24] Myers T. A. Crowther J. H. (2007). Sociocultural pressures, thin-ideal internalization, self-objectification, and body dissatisfaction: could feminist beliefs be a moderating factor? *Body Image* 4 296–308. 10.1016/j.bodyim.2007.04.001 18089276

[B25] Radloff L. S. (1977). The CES-D scale: a self-report depression scale for research in the general population. *Appl. Psychol. Measur.* 1 385–401. 10.1177/014662167700100306 26918431

[B26] Rosenberg M. (1965). Rosenberg self-esteem scale (RSE). Acceptance and commitment therapy. *Measur. Pack.* 61:52 10.1037/t01038-000

[B27] Stice E. Marti C. N. Durant S. (2011). Risk factors for onset of eating disorders: evidence of multiple risk pathways from an 8-year prospective study. *Behav. Res. Ther.* 49 622–627. 10.1016/j.brat.2011.06.009 21764035 PMC4007152

[B28] Thompson J. K. Heinberg L. J. (1999). The media’s influence on body image disturbance and eating disorders: we’ve reviled them, now can we rehabilitate them? *J. Soc. Issues* 55 339–353. 10.1111/0022-4537.00119

[B29] Thompson M. A. Gray J. J. (1995). Development and validation of a new body-image assessment scale. *J. Pers. Assess.* 64 258–269. 10.1207/s15327752jpa6402-6 7722852

[B30] Tiggemann M. McGill B. (2004). The role of social comparison in the effect of magazine advertisements on women’s mood and body dissatisfaction. *J. Soc. Clin. Psychol.* 23 23–44. 10.1521/jscp.23.1.23.26991

[B31] Vallieres E. F. Vallerand R. J. (1990). Traduction et validation canadienne-française de l’échelle de l’estime de soi de rosenberg. *Int. J. Psychol.* 25 305–316. 10.1080/00207599008247865

